# A Coumarin-Based Fluorescent Probe as a Central Nervous System Disease Biomarker

**DOI:** 10.3390/s141121140

**Published:** 2014-11-10

**Authors:** Ann-Chee Yap, Ummi Affah Mahamad, Shen-Yang Lim, Hae-Jo Kim, Yeun-Mun Choo

**Affiliations:** 1 Department of Chemistry, Faculty of Science, University of Malaya, Kuala Lumpur 50603, Malaysia; E-Mail: cady.yap@gmail.com; 2 Department of Medicine, Faculty of Medicine, University of Malaya, Kuala Lumpur 50603, Malaysia; E-Mails: ummiaffah@hotmail.com (U.A.M.); limshenyang@gmail.com (S.-Y.L.); 3 Department of Chemistry, Hankuk University of Foreign Studies, Yongin 449-791, Korea; E-Mail: haejkim@hufs.ac.kr

**Keywords:** fluorescent probe, fluorescence spectroscopy, mass spectrometry, homocysteine, methylmalonic acid, neurochemistry, chemoassay

## Abstract

Homocysteine and methylmalonic acid are important biomarkers for diseases associated with an impaired central nervous system (CNS). A new chemoassay utilizing coumarin-based fluorescent probe **1** to detect the levels of homocysteine is successfully implemented using Parkinson's disease (PD) patients' blood serum. In addition, a rapid identification of homocysteine and methylmalonic acid levels in blood serum of PD patients was also performed using the liquid chromatography-mass spectrometry (LC-MS). The results obtained from both analyses were in agreement. The new chemoassay utilizing coumarin-based fluorescent probe **1** offers a cost- and time-effective method to identify the biomarkers in CNS patients.

## 1. Introduction

**Scheme 1.**
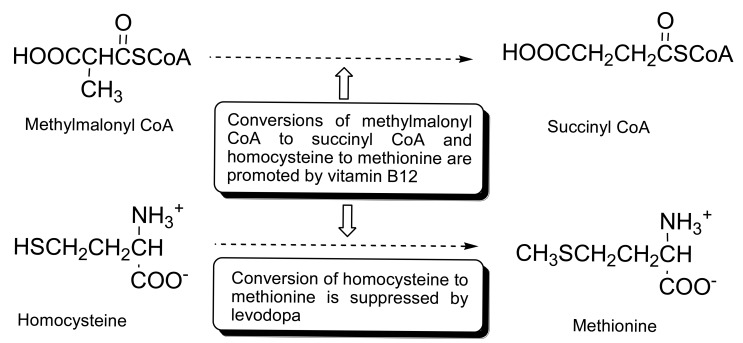
Conversion of homocysteine to methionine and methylmalonyl CoA to succinyl CoA.

The degeneration of the dopaminergic neurons accounts for the deficiency of dopamine which affects the central nervous system (CNS) [1–5]. Hence, the common treatment for the patients suffering from the central nervous system associated diseases, such as Parkinson's disease, Alzheimer's disease, chronic heart failure, multiple sclerosis, and epilepsy has focused on the control of the progression of the disease symptoms through the administration of levodopa (precursor of dopamine). Elevated levels of homocysteine and methylmalonic acid in the blood serum of Parkinson's disease (PD) patients are attributed to the inhibition of catabolism of homocysteine by levodopa treatment and/or deficiency of vitamin B12 of impaired nervous tissues. It has been reported that levodopa inhibits the catabolism of homocysteine to methionine in the folate pathway, which results in the elevation of homocysteine levels in the blood serum of the CNS disease patients who are on levodopa medication [6–9]. On the other hand, vitamin B12 is a cofactor in the conversion of homocysteine to methionine and methylmalonic acid to succinic acid in the folate pathway. Deficiency in vitamin B12 is often associated with the impaired nervous tissues and results in the elevation of homocysteine and methylmalonic acid levels in the patient serums. Hence, the levels of homocysteine, methylmalonic acid, and vitamin B12 in the serum of CNS disease patients are important biomarkers (Scheme 1). However, the use of vitamin B12 as a biomarker is less useful as compared to homocysteine or methylmalonic acid due to the instability of vitamin B12 and its low concentration in serum especially in the case of vitamin B12 deficiency [10,11].

**Scheme 2.**
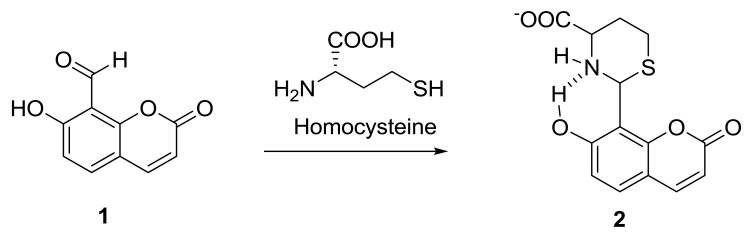
Reaction scheme of coumarin-based fluorescent probe **1** with homocysteine.

Previous reports on the detection of homocysteine, methylmalonic acid, and vitamin B12 focused on the use of immunoassays, HPLC-FD, GC-MS, and LC-MS methods [9–13]. Although the HPLC-FD method has the highest limit of detection (LOD), it has very poor selectivity as similar compounds present in a complicated biological matrix such as serum, tend to display similar retention times as the biomarkers (e.g., homocysteine, *etc.*) and hence may indicate the presence of higher levels of biomarkers that really present. On the other hand, the GC-MS method involves extra derivatization steps. Among these methods, the LC-MS method offers a compromise between sensitivity and selectivity and have been gaining steady popularity as the preferred choice. Chemoassays have been gaining popularity as more compounds with high selectivity are synthesized [14–22]. Most of these methods are demonstrated using standard mixtures spiked in pooled plasma as opposed to quantifying the biomarkers in the CNS patient's blood serum and comparing against the healthy subjects' blood serum. In the present study, we have evaluated the feasibility of a chemoassay using a coumarin-based fluorescent probe **1** (Scheme 2) to quantify homocysteine in the Parkinson Disease patients' blood serum and have established a rapid determination of homocysteine, methylmalonic acid, and vitamin B12 using LCMS. We have also successfully quantified the CNS disease biomarkers in the blood serum of Parkinson's disease patients with and without the levodopa treatment and compared against the healthy subjects.

## 2. Experimental Section

### 2.1. General

All the HPLC solvents were purchased from Merck Co. Ltd (Darmstadt, Germany) and used without further purification. The fluorescence data were recorded using Varian Cary Eclipse Fluorescence Spectrophotometer (Agilent, Santa Clara, CA, USA). The LCMS data were obtained on an Agilent 1200 series HPLC coupled to the Agilent 6530A Q-TOF mass spectrometer (Agilent, Santa Clara, CA, USA).

### 2.2. Fluorescence Spectroscopy Study

#### 2.2.1. Preparation and Fluorescence Measurement of Standards in Serum Matrix

DL-Homocysteine (H4628) and pooled human serum (H4522) were purchased from Sigma-Aldrich (St. Louis, MO, USA). The coumarin-based fluorescent probe **1** was prepared as described in the literature [23,24]. Standard samples containing 5 μL homocysteine were added to 145 μL pooled human serum to give final a volume of 150 μL. The precipitation of serum protein was achieved by incubating the mixtures with 150 μL of cold acetonitrile. The standard mixtures were then centrifuged for 20 min and 200 μL of the supernatant was pipetted out and added to 40 μL (100 μM) of fluorescent probe **1** and 160 μL of ultrapure water. The mixtures were incubated at room temperature for 2 h to ensure the complete binding of homocysteine to the fluorescent probe **1**. The final concentration of the standard mixtures are 10, 20, 50, 100, 150, 200, and 250 μM. 300 μL of the mixtures were added to 2700 μL and placed in the cuvette for fluorescence measurements (excitation frequency 365 nm, emission frequency 450 nm, Varian Cary Eclipse Fluorescence Spectrophotometer) [23,24].

#### 2.2.2. Preparation and Fluorescence Measurement of Serum Samples

The sera were obtained from PD patients from the University Malaya neurology clinic. The study was approved by the institutional ethics committee and all participants gave written informed consent. Serum samples (150 μL) were added with an equal amount of cold acetonitrile (150 μL). The mixtures were then treated and analyzed with fluorescence spectroscopy in similar the manner as described above in Section 2.2.1.

### 2.3. LCMS Study

#### 2.3.1. Preparation of Standards in Serum Matrix

Methylmalonic acid (99%, M54058) and vitamin B12 (V2876) were purchased from Sigma-Aldrich (St. Louis, MO, USA). Standard samples containing mixtures of homocysteine, methylmalonic acid, and vitamin B12 were prepared using 80 μL of pooled human serum and ultrapure water as solvent with a final volume of 200 μL. The precipitation of serum protein was achieved by incubating the mixtures with 200 μL of cold acetonitrile. The standard mixtures were then centrifuged for 20 min and 200 μL of the supernatant was pipetted out for LCMS analysis. The final concentrations of the standard mixtures were 0.1, 0.5, 1, 5, and 10 ppm of each homocysteine, methylmalonic acid, and vitamin B12.

#### 2.3.2. Preparation of Serum Samples

The sera were obtained from PD patients from the University Malaya neurology clinic. Serum samples (200 μL) were added with an equal amount of cold acenonitrile (200 μL). The mixtures were then treated and analyzed with LCMS in the similar manner as described above in Section 2.3.1.

#### 2.3.3. LC-MS Quantification of Homocysteine, Methylmalonic Acid, and Vitamin B12

An Agilent Zorbax SB-Aq column (2.1 × 100 mm, 3.5 μm) was used for the separation of the homocysteine, methylmalonic acid, and vitamin B12. The mobile phases used were 20 mM ammonium acetate and 0.1% formic acid in ultrapure water (A) and acetonitrile (B), with a total of 14 min run (0–2 min: 5% B isocratic gradient; 2–5 min: 5% to 80% B; 5–7 min: 80% B isocratic gradient; 7–7.5 min: 80% to 5% B; 7.5–14 min: 5% B isocratic gradient). The flow rate was 0.2 mL·min^−1^, column temperature was 40 °C, sample temperature was 5 °C, and injection volume was 30 μL. The samples were analyzed in triplicates with homocysteine and vitamin B12 in ESI-positive mode ([M + H]^+^ = 136.0427; 1.77 min) and ([M + 2H]^2+^ = 678.2910; 9.58 min), respectively, and methylmalonic acid in ESI-negative mode ([M − H]^−^ = 117.0193, 1.68 min). The data obtained from the LC-MS analysis were processed using Agilent MassHunter software.

## 3. Results and Discussion

### 3.1. Fluorescence Analysis

The coumarin-based fluorescent probe **1** was synthesized according to the literature and is highly selective towards the homocysteine and cysteine [23,24]. Compound **1** is non-fluorescent and has two important features, *i.e.*, the coumarin core structure which is the fluorescent signal unit and an aldehyde substituent which is the reaction site. The carbonyl group of the fluorescent probe **1** reacts selectively with homocysteine or cysteine to produce a strongly fluorescent six or five membered ring compounds **2**, respectively. The stability of the six/five membered rings formed in the reaction between the probe **1** and homocysteine or cysteine is attributed to the nucleophilicity of both the nitrogen and sulfur atoms. Hence, other amino acids which are lacking the sulfur atom will not be able to form the six/five membered rings and will not result in any fluorescence activities.

**Figure 1.**
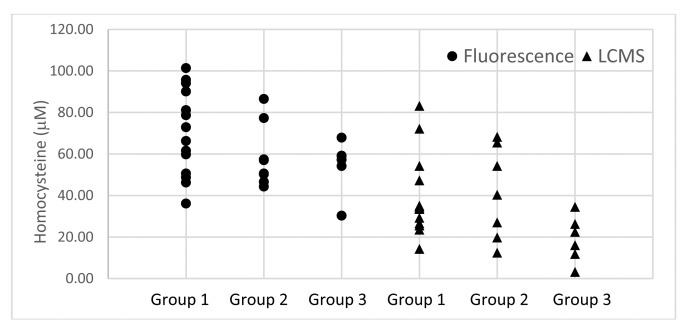
Homocysteine level measured by the fluorescence and LC-MS methods in PD patient sera (Group 1—PD patients not treated with levodopa; Group 2—PD patients treated with levodopa; Group 3—Healthy subjects).

The standard calibration curve was prepared by measuring the emission intensity of standard samples containing 10, 20, 50, 100, 150, 200, and 250 μM of homocysteine using fluorescence spectroscopy. Parkinson's disease patients attending regular outpatient visits at the University of Malaya neurology clinic from October 2010 to October 2011 were invited to participate in this study [25]. Sera were obtained from three groups of individuals: *i.e.*, patients who had not been treated with levodopa (Group 1; 14 subjects), patients treated with levodopa (Group 2; eight subjects), and control (Group 3; six healthy subjects). All the patients had been diagnosed with Parkinson's disease for between 1 to 10 years and were not on vitamin B12 supplementary medication. The sera were treated as described in the Experimental Section and the results of the analysis are summarized in Table 1 and Figure 1.

### 3.2. Fluorescence Results

The optimum emission frequency was determined in earlier studies to be 450 nm [23,24] and homocysteine and the coumarin-based fluorescent probe **1** were allowed to incubate for 2 h at room temperature to allow complete binding between the two materials. Standard mixtures containing 10, 20, 50, 100, 150, 200, and 250 μM of homocysteine were prepared and the emission intensities were measured to obtain a standard calibration curve. The blood sera from patients were treated with coumarin-based fluorescent probe **1** and subjected to fluorescence assay. The results (Figure 1 and Table 1) showed that the levels of homocysteine for the three test groups range from 36.12–101.38, 44.33–86.52, and 30.28–67.87 μM, corresponding to Groups 1, 2, and 3, respectively. The average concentrations of homocysteine in the blood sera of Group 1–3 are 70.24, 58.74, and 54.63 μM, respectively, showing a downward trend with the highest level of homocysteine in the non-levodopa treated patients (Group 1) and the lowest in the healthy subjects (Group 3), with the levodopa treated patients in the middle (Group 2).

### 3.3. LCMS Analysis

The levels of homocysteine, methylmalonic acid, and vitamin B12 in the serum of PD patients are important biomarkers with diverse characteristics and chemical properties. Homocysteine and methylmalonic acid are highly hydrophilic small molecules with very short retention times, while the larger vitamin B12 has a longer retention time. In addition, both homocysteine and vitamin B12 ionized better in ESI-positive mode while methylmalonic acid ionized in the ESI-negative mode. Standard mixtures in ultrapure water and human serum containing homocysteine, methylmalonic acid, and vitamin B12 were prepared for each compound with final concentrations of 0.1, 0.5, 1, 5, and 10 ppm, respectively, to investigate the method robustness and the matrix effects. The samples were analyzed in a 14 min gradient run in both the positive- and negative-mode. Homocysteine, methylmalonic acid, and vitamin B12 (detected as doubly charged species) were observed at 1.77 min for [M + H]^+^ = 136.0427, 1.68 min for [M − H]^−^ = 117.0193, and 9.58 min for [M + 2H]^2+^ = 678.2910, respectively (please refer to Supplementary Information).

### 3.4. LC-MS Results

**Figure 2.**
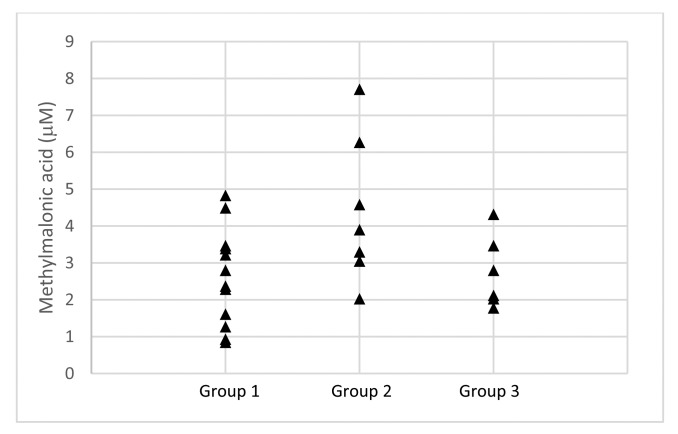
Methylmalonic acid level measurement by LCMS in PD patient sera (Group 1—PD patients not treated with levodopa; Group 2—PD patients treated with levodopa; Group 3—Healthy subjects).

The blood sera from the three groups described in the above section were subjected to LC-MS analysis. The patients' sera were treated as described in the Experimental Section and the results of the analysis are summarized in Table 1, Figures 1 and 2. The healthy subjects (Group 3) have homocysteine and methylmalonic acid levels ranging from 3.18–34.51 and 1.78–4.32 μM, with average values of 19.06 and 2.75 μM, respectively. GROUP 1 (not treated with levodopa) and Group 2 (treated with levodopa) patients have homocysteine and methylmalonic acid levels ranging from 14.26–83.16 μM and 0.85–4.83 μM, and 12.44–68.28 μM and 2.03–7.71 μM, respectively. The average levels of homocysteine and methylmalonic acid in Groups 1 and 2 are 38.48 and 2.46 μM, and 41.01 and 4.11 μM, respectively. Most of the PD patients showed higher level of homocysteine in blood sera when compared to that of the control Group 3.

We were unable to detect vitamin B12 in the sera for two reasons, firstly, the concentration of the vitamin B12 level in blood serum was very low, well below the detection limit of the LC-MS system; secondly, the instability of vitamin B12. Despite these setbacks on vitamin B12 quantification in the serum samples (e.g., Parkinson's disease patient's and healthy subject's serums), we have indeed demonstrated that the LC-MS method can be used to detect these characteristically diverse compounds based on the standard mixtures prepared from pooled human serum. With the rapid improvements on the detection limit of LC-MS, we anticipate that the method discussed here will present a platform for future analysis on such samples.

### 3.5. Comparison of Fluorescence and LC-MS Analysis Results and Methodology

The results for the levels of homocysteine in blood serum obtained by the fluorescence and LC-MS methods are shown in Figure 2 and Table 1. The overall homocysteine levels in all the groups, *i.e.*, Groups 1–3 obtained from the fluorescence method were higher than those from the LC-MS method. A possible explanation to this observation may be due to the thiol-group containing contaminants in the samples used in the fluorescence analysis, which are able to bind to probe **1**. The LC-MS samples were analyzed directly without dilution after sample treatment, while the fluorescence samples were diluted 10 times after sample treatment prior to analysis. The fluorescence method indicated that the levels of homocysteine in Group 1 ranged from 36.12–101.38 μM with an average value of 70.24 μM, while the LC-MS method indicated lower levels, *i.e.*, 14.29–83.16 μM and 38.48 μM, respectively. Similar observations were indicated in the Groups 2 and 3 results with homocysteine level ranges of 44.33–86.52 and 30.28–67.87 μM, and average values of 58.74 and 54.63 μM, respectively, obtained from the fluorescence analysis, and homocysteine level ranges of 12.44–68.28 and 3.18–34.51 μM, and average values of 41.01 and 19.06 μM, respectively, were obtained from the LC-MS analysis. However, the trends of the homocysteine levels appear to be similar in both the fluorescence and the LC-MS analysis, *i.e.*, highest levels of homocysteine were observed in Group 1 followed by Group 2 and lowest in Group 3.

The overall cost and preparation steps involved in the fluorescence analysis made its more efficient when compared to LC-MS analysis. The cost of consumables such as HPLC grade solvents and columns, and the LC-MS system is much higher than that required in the fluorescence analysis. Although the fluorescence analysis requires longer sample preparation time, *i.e.*, binding period between the fluorescent probe to the homocysteine, the additional sample preparation time in the fluorescence analysis is off-set by the much shorter overall analysis period when compared to that of LC-MS analysis. In addition, the fluorescence analysis method posed very little operational obstacles while the LC-MS analysis requires much more troubleshooting and careful maintenance, especially in the current analysis which deals with a complex serum matrix. The selectivity in the LC-MS analysis method is much broader than that of the fluorescence analysis. In the LC-MS analysis utilizing the QToF-MS detector, all the metabolites in the samples are recorded and the data can be revisited should a need arise. However, the fluorescence analysis method described in the present study is limited to detecting homocysteine. The sensitivity of both analysis methods appear to be comparable. The major inconvenience of the fluorescence analysis method is the access to the coumarin-based fluorescent probe **1** which is not commercially available at present.

## 4. Conclusions

In conclusion, we have evaluated the application of coumarin-based fluorescent probe **1** to determine the level of homocysteine, an important biomarker in the Parkinson's disease patients using the simple and cost effective fluorescence spectroscopy technique. In addition, we have also established a rapid determination of homocysteine and methylmalonic acid (another important biomarker) in the serum of Parkinson's disease patients using LC-MS. Although the fluorescent analysis method indicated higher levels of homocysteine in the PD patients when compared to that of the LC-MS analysis method, the trend of results are similar with the highest homocysteine levels recorded in Group 1 (PD patients not treated with levodopa) and the lowest homocysteine levels in Group 3 (healthy subject), with Group 2 (PD patients treated with levodopa) in the middle. The overall cost and ease of analysis suggest that the fluorescence analysis method is more effective compared to the LC-MS analysis.

Elevated levels of homocysteine and/or methylmalonic acid in blood serum are important indicators to assist in the identification of patients suffering from neurological and psychiatric diseases such as Parkinson's disease, Alzheimer's disease, chronic heart failure, multiple sclerosis, depression, epilepsy, and also vitamin B12 deficiency. The methods presented herein allow for the rapid identification of the homocysteine and methylmalonic acid levels in the blood serum, able to distinguished between the healthy subjects and PD patients, and can be extended to other patients suffering from the above mentioned diseases. The methods presented herein will hopefully be exploited for standard routine analysis in a high-throughput clinical setting.

## Supplementary Material



## Figures and Tables

**Table 1.** Comparison of data from fluorescence and LC-MS detection of homocysteine and methylmalonic acid in PD patient sera.

	**Homocysteine (Fluorescence) (μM)**	**Homocysteine (LCMS) (μM)**	**Methylmalonic Acid (LCMS) (μM)**
**Group 1**			

Average	70.24	38.48	2.46
Standard deviation	20.68	19.40	1.29
Median	69.58	33.66	2.33
Max.	101.38	83.16	4.83
Min.	36.12	14.29	0.85

**Group 2**			

Average	58.74	41.01	4.11
Standard deviation	12.76	20.65	2.01
Median	53.79	40.36	3.60
Max.	86.52	68.28	7.71
Min.	44.33	12.44	2.03

**Group 3**			

Average	54.63	19.06	2.75
Standard deviation	12.76	11.10	0.98
Median	58.12	19.26	2.46
Max.	67.87	34.51	4.32
Min.	30.28	3.18	1.78
