# Supplementary material for: A Coumarin-Based Fluorescent Probe as a Central Nervous System Disease Biomarker
Source: Sensors (Basel). 2014 Nov 10;14(11):21140–50. doi: 10.3390/s141121140 (PMC4279527; doi:10.3390/s141121140)
Supplement: Supplementary file 1 [file sensors-14-21140-s001.pdf]

OPEN ACCESS

sensors

ISSN 1424-8220
www.mdpi.com/journal/sensors

Supplementary Information

Coumarin-Based Fluorescent Probe for Central Nervous System Disease Biomarker. *Sensors* 2014, *14*, 21140-21150

Ann-Chee Yap [1], Ummi Affah Mahamad [2], Shen-Yang Lim [2], Hae-Jo Kim [3] and Yeun-Mun Choo [1,*]

[1] Department of Chemistry, Faculty of Science, University of Malaya, Kuala Lumpur 50603, Malaysia; E-Mail: cady.yap@gmail.com
[2] Department of Medicine, Faculty of Medicine, University of Malaya, Kuala Lumpur 50603, Malaysia; E-Mails: ummiaffah@hotmail.com (U.A.M.); limshenyang@gmail.com (S.-Y.L.)
[3] Department of Chemistry, Hankuk University of Foreign Studies, Yongin 449-791, Korea; E-Mail: haejkim@hufs.ac.kr

* Author to whom correspondence should be addressed; E-Mail: ymchoo@um.edu.my; Tel.: +603-7967-4237; Fax: +603-7967-4193.

List of Tables and Figures:

Table S1. Fluorescence data of homocysteine in standard sample.
Table S2. LC-MS measurements of standard samples.
Table S3. Fluorescence and LC-MS data of homocysteine and methylmalonic acid in PD patient serums.
Figure S1. Standard calibration curve of homocysteine from fluorescence study.
Figure S2. LC-MS EIC of homocysteine, methylmalonic acid, and vitamin B12.
Figure S3. Standard calibration curve of homocysteine, methylmalonic acid, and vitamin B12 from LC-MS study.

Table S1. Fluorescence data of homocysteine in standard sample.

Standard Concentration (µM)	Fluorescence Intensity (au)	Detected Concentration (µM)	Recovery (%)
10	74.49 ± 0.84	7.32	73
20	84.38 ± 1.44	27.36	137
50	96.05 ± 0.91	51.03	102
100	113.46 ± 6.25	86.32	86
150	148.81 ± 11.48	157.97	105

Table S2. LC-MS measurements of standard samples.

Compound	R^2	Standard Concentration (μM or nM)	Standard Concentration (ppm)	Detected Concentration	Recovery (%)
Homocysteine	0.9996	0.741 μM	0.1	0.516 ± 0.232 μM	70
		3.71 μM	0.5	2.319 ± 0.086 μM	63
		7.41 μM	1	6.420 ± 0.678 μM	87
		37.1 μM	5	34.481 ± 0.430 μM	93
		74.1 μM	10	69.770 ± 0.382 μM	94
Methymalonic acid	0.9985	0.847 μM	0.1	0.489 ± 0.095 μM	58
		4.24 μM	0.5	3.248 ± 0.160 μM	77
		8.47 μM	1	9.205 ± 0.269 μM	109
		42.4 μM	5	41.198 ± 0.972 μM	96
		84.7 μM	10	85.924 ± 1.456 μM	101
Vitamin B12	0.9995	0.0737 nM	0.1	0.119 ± 0.005 nM	161
		0.369 nM	0.5	0.318 ± 0.030 nM	86
		0.737 nM	1	0.666 ± 0.111 nM	90
		3.69 nM	5	3.375 ± 0.044 nM	91
		7.37 nM	10	6.579 ± 0.016 nM	89

Table S3. Fluorescence and LC-MS data of homocysteine and methylmalonic acid in PD patient sera.

	No.	Homocysteine (Fluorescence) (µM)	Homocysteine ± Std Dev (LC-MS) (µM)	Methylmalonic Acid ± Std Dev (LC-MS) (µM)
Group 1	1	36.12	25.55 ± 1.33	2.37 ± 0.08
	2	95.69	34.66 ± 1.11	1.27 ± 0.01
	3	46.27	83.16 ± 4.52	1.61 ± 0.17
	4	72.91	23.55 ± 1.18	2.80 ± 0.17
	5	90.11	29.25 ± 2.07	0.93 ± 0.01
	6	94.09	33.47 ± 6.81	4.83 ± 0.08
	7	66.25	26.59 ± 6.37	2.29 ± 0.08
	8	48.79	47.25 ± 3.04	1.27 ± 0.17
	9	59.79	14.29 ± 0.30	3.47 ± 0.17
	10	61.57	54.28 ± 1.48	4.49 ± 0.01
	11	81.11	72.13 ± 1.11	1.61 ± 0.25
	12	50.61	35. 10 ± 2.07	0.85 ± 0.08
	13	78.64	33.84 ± 1.56	3.39 ± 0.08
	14	101.38	25.62 ± 2.81	3.22 ± 0.25
Average		70.24	38.48	2.46
Standard deviation		20.68	19.40	1.29
Median		69.58	33.66	2.33
Max.		101.38	83.16	4.83
Min.		36.12	14.29	0.85
Group 2	1	86.52	68.28 ± 1.63	3.05 ± 0.17
	2	44.33	12.44 ± 2.89	2.03 ± 0.01
	3	50.57	65.54 ± 2.07	3.30 ± 0.17
	4	57.01	40.36 ± 1.41	2.03 ± 0.08
	5	57.37	54.28 ± 3.63	3.90 ± 0.25
	6	77.29	19.77 ± 0.52	4.58 ± 0.17
	7	46.57	40.36 ± 0.22	6.27 ± 0.17
	8	50.25	27.03 ± 2.22	7.71 ± 0.08
Average		58.74	41.01	4.11
Standard deviation		12.76	20.65	2.01
Median		53.79	40.36	3.60
Max.		86.52	68.28	7.71
Min.		44.33	12.44	2.03
Group 3	1	30.28	3.18 ± 0.52	2.03 ± 0.01
	2	57.22	11.85 ± 0.81	1.78 ± 0.08
	3	59.14	16.07 ± 0.96	2.12 ± 0.08
	4	67.87	26.29 ± 1.56	4.32 ± 0.08
	5	54.22	22.44 ± 0.44	3.47 ± 0.08
	6	59.03	34.51 ± 1.48	2.80 ± 0.08
Average		54.63	19.06	2.75
Standard deviation		12.76	11.10	0.98
Median		58.12	19.26	2.46
Max.		67.87	34.51	4.32
Min		30.28	3.18	1.78

Figure S1. Standard calibration curve of homocysteine from the fluorescence study.

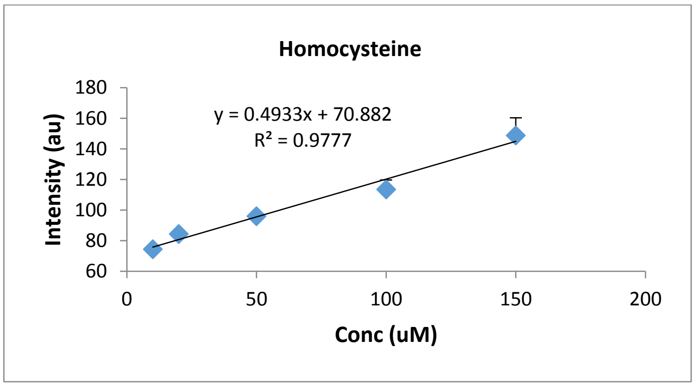

Figure S2. LC-MS EIC of homocysteine, methylmalonic acid, and vitamin B12.

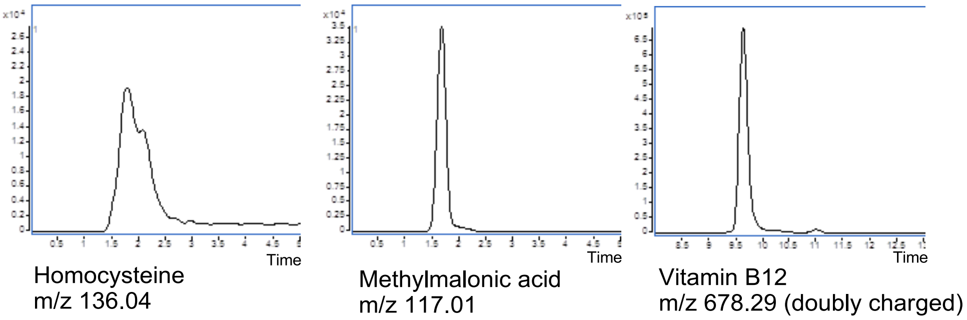

Figure S3. Standard calibration curve of homocysteine, methylmalonic acid, and vitamin B12 from LC-MS study.

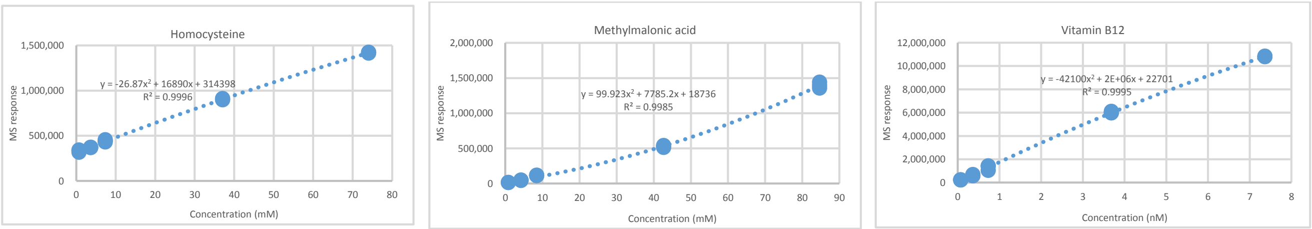

© 2014 by the authors; licensee MDPI, Basel, Switzerland. This article is an open access article distributed under the terms and conditions of the Creative Commons Attribution license (http://creativecommons.org/licenses/by/4.0/).
